# European principles of inhibitor management in patients with haemophilia

**DOI:** 10.1186/s13023-018-0800-z

**Published:** 2018-04-27

**Authors:** P. L. F. Giangrande, C. Hermans, B. O’Mahony, P. de Kleijn, M. Bedford, A. Batorova, J. Blatný, K. Jansone, on behalf of the European Haemophilia Consortium (EHC) and the European Association for Haemophilia and Allied Disorders (EAHAD), J. Astermark, A. Bok, M. Crato, R. d’Oiron, A. Dougall, K. Fijnvandraat, S. Grønhaug, V. Jiménez-Yuste, M. Jokić, S. Lobet, B. Nolan, F. Peyvandi, A. Ryan

**Affiliations:** 1 European Haemophilia Consortium, Rue de l’Industrie, B-1000 Brussels, Belgium; 2 University of Oxford, Oxford, UK; 3 Haemostasis and Thrombosis Unit, Division of Haematology, Cliniques Universitaires Saint-Luc, Brussels, Belgium; 4 Trinity College, Dublin, Ireland; 5 Department of Rehabilitation, Nursing Science and Sports, University Medical Center Utrecht, Utrecht, The Netherlands; 6 Canterbury Christ Church University, Kent, UK; 7 National Hemophilia Center, Department of Hematology and Transfusion Medicine, School of Medicine of Comenius University and University Hospital, Bratislava, Slovakia; 8 Children’s University Hospital Brno, Brno, Czech Republic

**Keywords:** Haemophilia, Guidelines, Inhibitors, Factor VIII, Factor IX, Bypassing agents, Immune tolerance

## Abstract

**Background:**

In spite of recent major advances in the understanding and treatment of inhibitor development in patients with haemophilia, multidisciplinary management of many of these patients remains suboptimal and highly heterogenous across Europe.

**Methods:**

Following a series of multidisciplinary meetings and a review of the literature, the European haemophilia community of health professionals and patients jointly defined practical optimum standards for ensuring and harmonizing treatment and care for patients with an inhibitor.

**Results:**

Ten complementary principles for the management of inhibitors in haemophilia have been developed, emphasizing the importance and benefits of a centralized, multidisciplinary, expert and holistic approach.

**Conclusions:**

This document will serve as a benchmark to improve the multidisciplinary and practical management of patients with inhibitor. Implementation and adherence to each of these principles should have a major positive impact on the management and outcomes of patients developing an inhibitor.

## Background

Haemophilia A and B are inherited bleeding disorders caused by deficiencies of coagulation factors VIII (FVIII) and IX (FIX) respectively [1]. The hallmark of the severe phenotype is recurrent and spontaneous haemarthrosis, which can eventually result in arthropathy, impaired mobility and chronic pain.

Inhibitor development is an immunological reaction of the body to exogenous factor VIII or IX. It is now the most serious complication in haemophilia, following the effective elimination of the risk of transmission of blood borne pathogens such as HIV or hepatitis C. The presence of circulating neutralizing antibodies against FVIII or FIX inactivates infused coagulation factor proteins, thus impairing their clinical efficacy [2]. Inhibitors are classified into low (<5Bethesda Units [BU])/ml) and high (>5BU) titre. Antibodies may be further classified as either low- or high-responding, according to whether the titre rises significantly after treatment with factor VIII. In cases where a patient with non-severe hemophilia develops inhibitors against factor VIII, endogenous clotting factor may also be inactivated. This can result in the change of a previously moderate/mild disorder into a severe phenotype [3].

## Discussion

While the importance of inhibitor management in the care of people with haemophilia was highlighted almost a decade ago in the European Principles of Care [4], that document focused principally on medical treatment and eradication of inhibitors. This document proposes ten principles of comprehensive inhibitor management, which involve all aspects of care and treatment (Table 1).

**Table 1:** Ten European Principles of Inhibitor Management

1. Awareness of the incidence of inhibitors and risk factors throughout life	
2. Early recognition and accurate diagnosis	
3. Optimal organization of care and communication between all stakeholders	
4. Haemostatic treatment with bypassing agents in inhibitor patients	
5. Inhibitor eradication by immune tolerance induction (ITI) therapy	
6. Access to, and optimal preparation for, surgery and other invasive procedures	
7. Provision of specialist nursing care	
8. Provision of tailored physiotherapy care and monitoring	
9. Access to psychosocial support	
10. Involvement in research and innovation	

## Methods

This document is the result of an initiative of healthcare professionals and patients representing the European Association for Haemophilia and Allied Disorders (EAHAD) and the European Haemophilia Consortium (EHC). Following a series of multidisciplinary meetings with European experts in haemophilia care and a review of publications in the field, ten principles for the multidisciplinary management of inhibitors in haemophilia have been developed. It is hoped that this document will serve as a benchmark to improve the management of patients with inhibitors.

### Awareness of the incidence of inhibitors and risk factors across the life-span

The incidence of inhibitor development is approximately 30% amongst patients with severe haemophilia A [5], but significantly lower in patients with non-severe haemophilia at around 3–13% [6]. The incidence of inhibitor development amongst patients with haemophilia B is much lower than in haemophilia A, in the range of 1–6%. Whilst the definition of incidence relates to the number of patients who ever develop an inhibitor, the prevalence indicates the proportion of affected patients at a specific time point. The reported overall prevalence of inhibitors in haemophilia is 5–7%. This lower figure reflects the fact that inhibitors disappear in many patients, either spontaneously or after immune tolerance induction therapy.

Some patients are more likely to develop inhibitors due to two classes of specific risk factors-genetic or environmental. Genotype constitutes the principal risk factor, with gene deletions carrying the highest risk [7]. A positive family history and African-American ethnicity are also recognized risk factors [2]. The environmental risk factors are less clearly understood although there is some evidence that intensity of early treatment may be a risk factor in previously untreated patients (PUPs) [8]. A recent prospective randomized study documented a higher incidence of inhibitor development among PUPs treated with recombinant factor VIII compared to those treated with plasma-derived products which contained von Willebrand factor [9]. Change of treatment product (for example after a national tender) has not been shown to increase the risk of inhibitor development in previously treated patients (PTPs) [10]. Discussions on treatment options should take place with the parents of PUPs before starting treatment.

The first 50 exposure days (EDs) constitute the highest risk period for the development of inhibitors in PUPs with severe haemophilia after starting treatment. After this time, the risk falls off very considerably although a second but much smaller peak of inhibitor development has been observed in older patients in their 60s and beyond [11]. In patients with non-severe haemophilia A the risk of inhibitor development seems to be much lower, but when exposure days are taken into account the risk increases up to 13% at 100 ED [3]. The risk of inhibitor development does not reduce after 50 ED in patients with non-severe hemophilia and therefore lifelong vigilance is required in adults with non-severe haemophilia. Some *F8* genotypes seem to be associated with inhibitor development in these patients. The potential risk factors for individual patients should be considered, especially in the case of children who are about to embark upon treatment for the first time.

### Early recognition and accurate diagnosis

Early recognition and accurate diagnosis are essential for successful management. Inhibitors can be detected by performing a Bethesda assay with the Nijmegen modification [12]. Heat treatment of the test plasma improves the sensitivity of the test [13].

All previously untreated patients (PUPs) should be closely monitored and regularly screened for inhibitors. The first 50 exposure days (ED) represent the high-risk period. In severe haemophilia A patients, the initial screening should be performed every three exposure days (EDs) until 20 EDs; then every 10 EDs until 50 EDs; and later on at least two times a year until 150 EDs, after which the risk of inhibitor development is very low indeed. However, it is recommended that periodic screening should continue to be performed as part of the routine follow-up process on an annual basis [14]. Screening should also be performed prior to surgery or other invasive procedures and whenever the clinical response to conventional treatment is deemed inadequate. It is also recommended to perform inhibitor screening both before and some weeks after a switch in treatment product, although the risk associated with product switching appears to be very low [10]. It is essential for patients and treatment centres to keep records relating to product usage by individual patients in order to facilitate retrospective analysis.

Recent studies have highlighted the risk of inhibitor development after intensive treatment of patients with mild and moderate haemophilia [6]. In the light of this, it is recommended that patients with non-severe hemophilia A should be proactively screened for inhibitors approximately 6 weeks after surgery or treatment for a major bleed.

In the case of haemophilia B, it is recommended to identify the underlying genotype through DNA analysis in order to identify subjects potentially at higher risk for inhibitor development [15]. Inhibitor development in patients with haemophilia B is often associated with anaphylactic reactions. In cases where a high risk is identified (e.g. large gene deletion), or if the genotype is unknown, precautions may then be taken such as ensuring that the first 20 or so infusions are only given in a hospital setting with close monitoring.

If an initial inhibitor screening test based on a APTT (activated partial thromboplastin time) mix is positive, the result should be confirmed with Bethesda method (Nijmegen modification) on a fresh sample and the patient monitored closely in the meantime. Validated and reliable laboratory testing is an essential component of assessing and monitoring an inhibitor. Inhibitor detection and titration are essential for planning the optimal treatment and this requires a specialized laboratory. If the local laboratory is not able to identify or titrate inhibitors with confidence, a sample should be sent (or the patient referred) to a laboratory in an expert centre.

### Organization of care and communication between all stakeholders

The coordination and organization of multidisciplinary inhibitor management, and regular communication between the multidisciplinary care providers, are the key elements of effective management. Once diagnosed, every patient who has developed inhibitors should be followed up in one of the certified European Haemophilia Comprehensive Care Centres (EHCCC) or European Haemophilia Treatment Centres (EHTCs), where all the major decisions regarding treatment and care should be made [16]. Local care may be offered if the patient cannot be followed up in an expert centre, but only when there is effective communication with the EHCCC/EHTC. As a part of multidisciplinary care, peer support should be offered to those affected by inhibitors. Patients should be informed by the specialists at the treatment centres about patient organizations at the local and national levels.

### Haemostatic treatment with bypassing agents

In patients with low titre inhibitor (< 5 BU/ml), treatment with high doses of FVIII or FIX may stop the bleeding. However, this therapy is very likely to be ineffective in persons with high titre inhibitors (> 5 BU/ml) and such treatment may also subsequently boost the titre. These patients should be treated with bypassing agents in order to secure haemostasis.

There are currently only two licensed bypassing agents: activated prothrombin complex concentrate (aPCC) (FEIBA®, Shire) and recombinant activated factor VII (rFVIIa) (NovoSeven®, Novo Nordisk). Typical initial doses are 50–100 units/kg of FEIBA and 90 μg/kg of NovoSeven. Both products have been demonstrated to be similarly effective in treating patients with inhibitors [17]. It is advisable for haemophilia treatment centres to have both products readily available as some patients seem to respond to one agent better than the other [18].

NovoSeven® is regarded as the preferred product for treatment of bleeds before starting immune tolerance induction as FEIBA contains trace amounts of FVIII which may promote an anamnestic response and rise in inhibitor titre [19].

The use of desmopressin in patients with non-severe haemophilia will help to reduce the chance of inhibitor development [15].

Prophylactic treatment with bypassing agents should be considered in patients with persistent inhibitors who fail to achieve immune tolerance (see next section). This is now regarded as the optimal approach, as it has been demonstrated to decrease the number of bleeding episodes and increase quality of life [15].

### Inhibitor eradication by immune tolerance induction (ITI) therapy

Currently the only way to eradicate an inhibitor is through prolonged exposure to FVIII (or FIX) by frequent administration of these concentrates at high dosage. Successful Immune Tolerance Induction (ITI) suppresses the immune response and restores tolerance to exogenous FVIII or FIX and enables the treatment with these factors concentrates. Different modalities of ITI employ different dosages and intervals of factor administration, ranging from 12 hourly up 3 times a week. An international randomized study in haemophilia A compared outcome after treatment with 200 iu/kg factor VIII daily with 50 iu/kg three times weekly [20]. The overall outcome was similar for both regimes, with some 70% achieving immune tolerance with loss of inhibitor and a further 5% achieving a partial response. However, the time to achieve remission was significantly faster with the higher dose regime and, furthermore, there was an higher risk of breakthrough bleeds associated with the lower dose regime. It is usual to start ITI with the same product as the one being used when the inhibitor developed. If the patient begins treatment with a recombinant product and no response is evident after approximately 6 months, the possibility of switching to a plasma-derived won Willebrand factor containing concentrate should be considered. The most important prognostic indicator of a likely good response is an inhibitor titre of < 10 BU/ml. Interruption of regular infusions must be avoided as this has been shown to prejudice the final outcome [15]. Breakthrough bleeds should be treated with bypassing agents, given on demand or prophylactically in selected patients. The likelihood of achieving tolerance in haemophilia B is much lower than in haemophilia A and ongoing treatment with factor IX concentrates may provoke nephrotic syndrome [15].

ITI is a demanding and resource-heavy treatment, and the patient (or parents) should receive detailed and reliable information about modalities, implications and success rate before initiation of ITI. An indwelling venous access device may need to be implanted. The multidisciplinary care team should carry out an assessment of patient’s suitability for ITI, taking into account commitment, stability and potential adherence of the patient.

In patients with non-severe hemophilia A, the inhibitor may disappear spontaneously as a result of the immunologically tolerizing effect of endogenous circulating FVIII. However, this does not imply that the patient is tolerant for FVIII concentrate and so an anamnestic reaction may occur when the patient is re-challenged with factor concentrate [21].

A plan should be in place for the management of those patients who fail to respond to ITI. One option is prophylaxis with bypassing agents. Typical initial regimes include 85 u/kg FEIBA® on alternate days and NovoSeven® 90 μg daily [15]. Regular consideration should be given to whether novel agents may be suitable for these patients, preferably within the setting of a clinical trial.

### Access to, and optimal preparation for, surgery and other invasive procedures

Patients with inhibitors are often denied surgery or any invasive procedures as these entail high cost and are also perceived to carry a significant risk of bleeding. However, there is now an extensive body of evidence proving that surgery may be carried out safely and effectively under cover of bypassing agents [22, 23]. This may also prove cost-saving in the long term.

The multidisciplinary care team should help the patient to reach a well-informed decision about surgery or other invasive procedure. Any invasive procedure will require the relevant expertise and consultation within the entire multidisciplinary team, as well as carefully anticipated logistics and treatment plan developed together with the patient. Surgery can be only performed if a proper rehabilitation programme, tailored to the individual patient, is in place.

Regular dental reviews are strongly recommended along with effective methods of preventative home care [24]. Regular check-ups and timely preventative interventions will considerably reduce the development of dental disease and the risks and costs of dental procedures. Local fibrin sealants and anti-fibrinolytic agents are particularly helpful in reducing blood loss duringdental surgery.

### Provision of specialist nursing care

Provision of high quality specialist nursing care for haemophilia patients with inhibitors is of the utmost importance as the nurse has a central role in organization and the ongoing management of the condition [25]. This consists of coordinating input from different members of the multidisciplinary care team and interagency liaison when required, this being especially important with younger patients who are in early education. A key nursing responsibility is the administration of replacement factor and bypassing agents through both peripheral venous access and central venous access device. This incorporates their own practice but also training for self-treatment, and the maintenance of safe standards of administration in order to prevent avoidable risks such as infection [26]. The nurse offers support throughout the inhibitor management process, assessing coping abilities and adherence, and gains insight into the personal situation/individual needs of each patient and their family whilst acting as their main contact point for the multidisciplinary care team. The development of an inhibitor affects the whole family, especially when diagnosed in a young child, so additional support should be offered to the family to aid effective functioning [27]. This may include facilitating peer support by other individuals/families more experienced at living with an inhibitor, and signposting to national/local haemophilia patient organisations, as well as assessment of the patient for referral to other health and social professionals. The nurse plays an important role in evaluating the patients’ (and family members’) understanding of inhibitors and periodically educating them regarding the latest developments in inhibitor care in order to facilitate their informed choice and consent to treatment.

### Provision of tailored physiotherapy care and monitoring

Provision of physiotherapy is focused on maintaining optimal musculoskeletal function. In patients with inhibitors, especially children, bleeds are more difficult to control. Assessment and management of bleeds requires a careful balance of rest and activity; often with slower progress than with inhibitor-free patients [28]. If optimal functional recovery after every bleed is not achieved, both in joint [29] and muscle bleeds [30], the long-term consequences will be significant.

Physical monitoring requires regular measurement of body shape and function as well as activities. Inhibitor patients should have easy access to different musculoskeletal experts (occupational therapists, rehabilitation specialists and orthopaedic surgeons) within the multidisciplinary team as indicated by their needs.

Physiotherapy involvement should be flexible and carefully tailored to the individual patient, ensuring haemostatic cover at all times. The physiotherapist and haematologist should maintain regular contact with each other, to align the timing of factor infusions with more advanced exercises.

The rehabilitation of inhibitor patients after serious trauma or surgery is more complex and may require a different and slower approach than in non-inhibitor patients. The goals of rehabilitation should be tailored and function-focused and should be steered by the musculoskeletal expert who can communicate fully the aims with the rest of the team, as well as with the patients and his family.

### Access to psychosocial support

The treatment and care of haemophilia with inhibitors represents a major burden for the patient, as well as for the family and caregivers, and the immediate social network, e.g. school or work [31]. Elements of life which may be psychologically challenging for the patient and the family/caregiver are the unpredictability of bleed, successful transitioning to self-care, family life and reproductive choices, as well as pain and fear of pain. These may cause low self-esteem, anger and frustration, and subsequently anxiety and depression in patients and caregivers in the family [32]. Social challenges, such as social isolation and exclusion due to frequent absence from school or work, poor access to insurance etc. increase the psychological burden. Support for patients and their families should be provided: this should encompass psychological support as well as advice on employment and social security benefits. Members of the multidisciplinary care team should be proactive and refer patients to a social worker and/or psychologist when any needs are identified. Local and national patient organisations are also a very good source of advice and support.

### Involvement in research and innovation

Improvements in clinical outcomes for inhibitor patients will increasingly depend on future research and innovation. It is highly desirable that patients with inhibitors are registered with treatment centres where innovations and contribution to research are accessible. National patient registers can help to identify eligible patients for relevant clinical trials.

Up-to-date information about the timeline and enrollment procedures of clinical trials is available on the website of the EUHANET project (www.haemophiliacentral.org), and should be further disseminated by the patient organisations.

Pharmacovigilance is an essential aspect of innovation in terms of patient safety [33]. Adverse event reporting through the European Haemophilia Safety Surveillance (EUHASS, www.euhass.org) platform should be encouraged by the healthcare professionals and patient organisations.

## Conclusions

It is hoped that this document will serve as a benchmark to improve the multidisciplinary and practical management of patients with haemophilia and inhibitors.

## Abbreviations

APTT: Activated partial thromboplastin time
BU: Bethesda Units
DNA: Deoxyribose nucleic acid
EAHAD: European Association for Haemophilia and Allied Disorders
ED: Exposure days
EHC: European Haemophilia Consortium
EHCCC: European Haemophilia Comprehensive Care Centre
EHTC: European Haemophilia Treatment Centre
HIV: Human Immunodeficiency virus
ITI: Immune tolerance induction
PTP: Previously treated patient
PUP: Previously untreated patient
